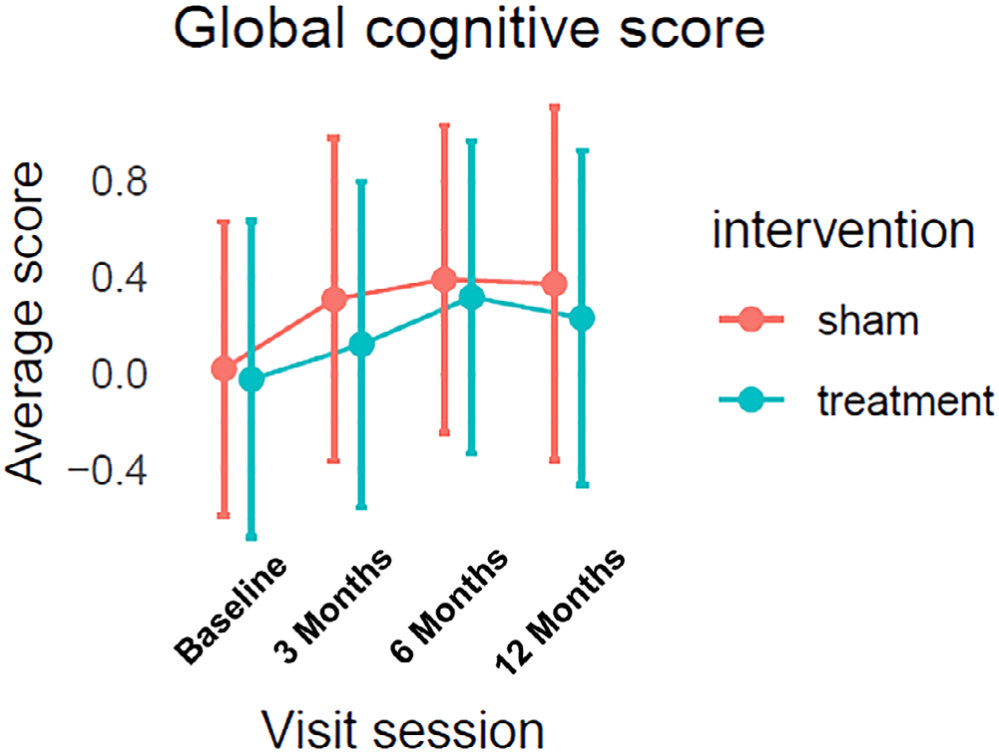# Results of a double‐blind sham‐controlled trial examining the effects of hyperbaric oxygen therapy on cognition and brain biomarkers

**DOI:** 10.1002/alz70861_108814

**Published:** 2025-12-23

**Authors:** Ori Benari, Ramit Ravona‐Springer, Lan Luo, Liangyuan Hu, Yael Mardor, Orit H. Lesman‐Segev, Sapir Golan, Abigail Livny, Maayan Harel, Ganit Almog, Shai Efrati, Amir Hadanny, Roy Sagi, Barbara B. Bendlin, Mary Sano, Michal Schnaider Beeri

**Affiliations:** ^1^ The Joseph Sagol Neuroscience Center, Sheba Medical Center, Ramat Gan, Ha Merkaz Israel; ^2^ Faculty of Medicine and Health Sciences, Tel Aviv University, Tel Aviv, HaMerkaz Israel; ^3^ Memory clinic, Sheba Medical Center, Tel Hashomer Israel; ^4^ The Joseph Sagol Neuroscience Center, Sheba Medical Center, Tel Hashomer Israel; ^5^ Rutgers School of Public Health, New Jersey, NJ USA; ^6^ Rutgers School of Public Health, Piscataway, NJ USA; ^7^ Faculty of Medical and Health Science, Tel Aviv University, Tel Aviv Israel; ^8^ The Advanced Technology Center, Sheba Medical Center, Ramat Gan, HaMerkaz Israel; ^9^ Department of Diagnostic Imaging, Sheba Medical Center, Tel Hashomer Israel; ^10^ The Sagol School of Neuroscience, Tel Aviv University, Tel Aviv Israel; ^11^ C. BIRD‐Clinical Brain Imaging R&D Center, Sheba Medical Center, Ramat Gan Israel; ^12^ Bar‐Ilan University, Ramat Gan Israel; ^13^ The Joseph Sagol Neuroscience Center, Sheba Medical Center, Ramat Gan Israel; ^14^ Sagol center for Hyperbaric Medicine & Research, Shamir (Assaf Harofeh) Medical Center, Beer Yaakov Israel; ^15^ Faculty of Medicine and Health Sciences, Tel Aviv University, Tel Aviv Israel; ^16^ Wisconsin Alzheimer's Disease Research Center, School of Medicine and Public Health, University of Wisconsin‐Madison, Madison, WI USA; ^17^ Icahn School of Medicine at Mount Sinai, New York, NY USA; ^18^ Herbert and Jacqueline Krieger Klein Alzheimer’s Research Center, Piscataway, NJ USA

## Abstract

**Background:**

Older adults with type 2 diabetes (T2D) and mild cognitive impairment (MCI) are at increased risk for dementia. Hyperbaric oxygen therapy (HBOT) has shown vascular and metabolic effects that may benefit brain health. This randomized, double‐blind, sham‐controlled trial assessed the impact of HBOT on cognitive function, cerebral blood flow (CBF), and glucose metabolism in this high‐risk group.

**Methods:**

A total of 155 participants were recruited and randomized to HBOT (*N* =77) and sham (*N* =78) between 2017 and 2023. Cognitive outcomes were assessed at baseline, 3, 6, and 12 months. Imaging (ASL‐MRI for CBF and FDG‐PET for glucose metabolism) was conducted at baseline, 3, and 12 months. The primary outcomes included global cognition (composite z‐score), CBF, and FDG‐PET. SUVR values were calculated using a global cortical ROI as the target region, normalized to the pons as the reference region. Secondary cognitive measures included domain‐specific cognition (executive functions and episodic memory) and CDR‐SOB. Analyses used linear mixed‐effects models under intent‐to‐treat (ITT) and per‐protocol (PP) approaches, without adjustment for multiple comparisons.

**Results:**

Across all timepoints, participants improved in cognition compared to baseline. At 3 months, global cognition and executive function significantly favored sham (*p* <0.05), with no sustained group differences at later timepoints (see Figure). Memory improved over time in both groups, with no significant group differences. There were no differences in CBF or FDG‐PET SUVR between groups. Sex and baseline cognition (median CDR‐SOB) had no significant effects on treatment response. PP analyses suggested lower SUVR at 3 months in the HBOT group across several brain regions (*p* ‐values: 0.014‐0.048). No group differences were seen in correlations between changes in cognition and CBF or SUVR. Adverse event analysis showed three times more serious adverse events (SAEs) in the HBOT group (*N* =25) comparing to sham (*N* =8), distributed across multiple organ systems. AEs distribution was similar between groups.

**Conclusions:**

HBOT did not improve cognitive or brain imaging outcomes compared to sham in older adults with T2D and MCI. A transient cognitive benefit was observed in the sham group immediately post‐intervention. The higher rate of SAEs in the HBOT group for this population with high comorbidities warrants further investigation.